# Prenatal diagnosis of HNF1b mutation allows recognition of neonatal dysglycemia

**DOI:** 10.1007/s00592-020-01641-2

**Published:** 2020-12-01

**Authors:** Fernanda Iafusco, Serena Meola, Carmine Pecoraro, Cristina Mazzaccara, Dario Iafusco, Nadia Tinto

**Affiliations:** 1 Department of Molecular Medicine and Medical Biotechnology, University “Federico II”, Naples, Italy; 2 CEINGE Advanced Biotechnology, Naples, Italy; 3 Nephrologist Unit, Santobono Children’s Hospital, Naples, Italy; 4 Department of Pediatrics, University of Campania “Luigi Vanvitelli”, Naples, Italy

**Keywords:** Prenatal diagnosis, Maturity onset diabetes of the young (MODY), Neonatal HNF1b dysglycemia

## Introduction

Maturity onset diabetes of the young (MODY) is the most common form of monogenic diabetes in Europe, affecting between 1 and 6% of diabetic patients. It comprises a group of heterogeneous genetic disorders characterized by early onset of diabetes (commonly before age 25), absence of autoimmunity, and beta-cell dysfunction. So far, mutations in 14 different genes involved in glucose homeostasis and pancreatic development [1] have been associated with this disease. Although it is an autosomal dominant disorder, de novo mutations should be taken into consideration in patients without a family history of diabetes. Most cases of MODY are due to mutations in *GCK, HNF1a, HNF4a* and *HNF1b*, previously known as MODY2, MODY3, MODY1, and MODY5, respectively. Among these, HNF1b is an active transcription factor that forms homodimers or heterodimers with HNF1a and plays a fundamental role in kidney development, nephron differentiation, and pancreatic growth and differentiation. Mutations in this gene lead to congenital anomalies of the kidney and urinary tract, genital malformations, pancreatic atrophy with endocrine and exocrine deficiency [2]. Diabetes usually onsets in early adulthood and frequently requires insulin treatment.

We present an unusual case of a prenatal diagnosis that revealed a mutation in the *HNF1b* gene responsible for neonatal hyperglycemia in a pregnant woman affected by X-linked ectodermal dysplasia.

## Case report

We describe the case of a 30-year-old pregnant woman affected by incontinentia pigmenti, a rare multisystemic ectodermal dysplasia with X-linked dominant inheritance. 16 weeks into her first pregnancy, she underwent amniocentesis to find out whether her fetus had inherited the disease. Although fetal DNA resulted negative for the maternal disease, the fetal anatomy ultrasound showed severe renal abnormalities, namely, increased echogenicity of the renal parenchyma and bilateral kidney enlargement. Accordingly, we performed a clinical exome analysis by Next Generation Sequencing (NGS) (Illumina Next-Seq500) including about 5000 disease-associated genes and limited the bioinformatics analysis to *HNF1b, PKD1, PKD2, PKHD1* genes associated with renal cysts to identify the genetic cause of the kidney alterations. A heterozygous nucleotide variant in the donor splicing site of intron 1 (c.344 + 2 T > *C*) of the *HNF1b* gene was identified. This mutation was also confirmed after birth by Sanger sequencing. Furthermore, a bioinformatics analysis (Alamuth Visual v.2.11.0—Alamut.interacrive-biosoftware.com) predicted that such type of mutation resulted in an mRNA splicing defect causing either exon 2 skipping or intron 1 retention. The mutation is reported in Human Gene Mutation Database (HGMD) (http://www.hgmd.cf.ac.uk.ac) in association with HNF1b/MODY. Molecular sequencing extended to the parents showed the absence of the variant, thereby indicating that the mutation was de novo.

**Fig.1:**
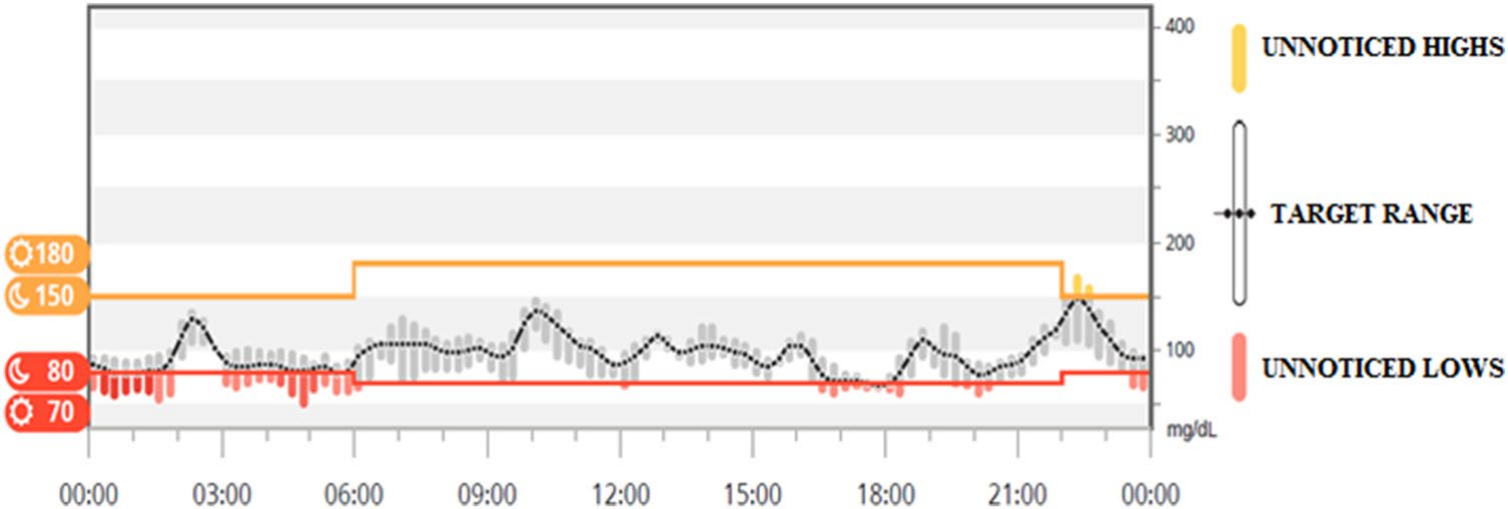
Continuous glucose monitoring (CGM) of the patient after 3 weeks from the birth

Although HNF1b-related diabetes most commonly develops in early adulthood (median age 20 years), we decided, nonetheless, to perform a careful blood glucose monitoring of the newborn. Interestingly, occasional glycosuria and capillary blood glucose, ranging between 65 mg/dL (3.61 mmol/L) and 212 mg/dL (11.78 mmol/L), were detected as early as the second week of life. Then, at 3 weeks, we monitored the infant’s interstitial fluid glucose levels for 5 consecutive days using a real-time continuous glucose monitoring (RT-CGM) (Dexcom G4) (2). We evidenced increased levels of post-prandial blood glucose and high glycemic variability (Fig. 1). In particular, blood glucose levels ranged from 80 to 150 mg/dL (4.44–8.33 mmol/L) during most of the monitoring time, whereas they were below 70 mg/dL (3.89 mmol/L) and above 150 mg/dL (8.33 mmol/L) for short periods of time.

Based on these test results, we diagnosed a "neonatal form of HNF1b dysglycemia”.

## Discussion

MODY is an autosomal dominant form of early-onset diabetes. Mutations in the *HNF1b* gene are responsible for the development of HNF1b/MODY, which is associated with distinctive clinical features, including pancreatic atrophy and renal disease [2, 3]. In this case report, we described an unusual prenatal diagnosis that revealed a mutation in the *HNF1b* gene in the fetus of a woman affected by incontinentia pigmenti. Additional laboratory testing, performed shortly after birth, led to a diagnosis of neonatal hyperglycemia.

The diagnostic process of such complex case greatly benefitted from the application of NGS. Indeed, as opposed to more traditional sequencing techniques, the advent of high-throughput NGS technology has greatly enhanced our understanding of genetic diseases, enabling researchers and clinicians to analyze multiple gene panels simultaneously in a cost-effective and time-saving manner. In our specific case, it was instrumental not only in diagnosing but also in elucidating the genetic causes and consequences of the condition.

In particular, although the fetus resulted negative for the maternal disease, it presented renal anomalies, as evidenced by the fetal ultrasound anatomy scan. Therefore, in an effort to clarify the possible causes of these abnormalities, we used NGS to identify the molecular defect related to these alterations. The analysis detected the presence of a variant in the *HNF1b* gene that encodes a transcription factor involved in the organogenesis of the kidneys, urinary tract, liver, and pancreas.

The rarity of our case lies in the fact that HNF1b/MODY is generally diagnosed in young adults, when disease-associated complications may already have developed. Affected patients may also have renal morphological anomalies (in particular cysts associated with alterations in renal function), morphological and functional anomalies of the exocrine pancreas and of the urogenital tract, and alterations in liver tests. The severity and the age of the onset of diabetes can vary greatly depending on the types of mutations involved in the *HNF1b* gene; further, the extent of kidney damage can also be equally heterogeneous*.*

The variant identified in our patient is known to alter mRNA splicing mechanisms with important consequences on the protein. In addition, exons 1–4 of the gene encode the dimerization and DNA binding domains of the protein. Mutations in these domains are predicted either to alter the ability of hetero/homodimer formation or to change the DNA targeting sequence with important consequences. In our patient, in addition to renal anomalies, we evidenced neonatal hyperglycemia, an event that is highly unusual at this age. To date, only three cases of *HNF1b* mutations associated with neonatal diabetes have been reported in the literature [2, 3].

The first two cases were reported by Yorifuji et al. They described a case of two siblings with the same heterozygous mutation of the *HNF1b* gene, but with discordant phenotypes: the first presented permanent neonatal diabetes mellitus, whereas the second presented polycystic and dysplastic kidneys that resulted in early renal failure [2].

The third case was reported by Beckers et al. The authors described the case of a newborn who received a 48 h intravenous insulin therapy to control an episode of hyperglycemia during parenteral feeding. However, at the age of 5, he developed permanent non-autoimmune diabetes. In addition, although he had no renal cysts, renal function progressively decreased [3].

Unlike these previous cases, in our patient the fetal genetic anomaly was detected in the prenatal period. It was such discovery that prompted us to monitor the glycemic changes associated with the *HNF1b* gene mutation as early as the baby’s first few days of life.

Evidence that hyperglycemia may be present immediately after birth confirms that in patients carrying *HNF1b* mutation the glycemic defect is due to neonatal insulin deficiency. Moreover, Hattersley et al. have observed that, among MODY patients, HNF1b/MODY newborns from normo-glycemic mothers are those with the lowest birth weight, compared with GCK/MODY or HNF1a/MODY newborns [4, 5]. This evidence confirms that insulin deficiency, which represents one of the main hormones implicated in fetal growth, is already present in the prenatal period. Indeed, in our case, the baby, whose mother was normo-glycemic, was nonetheless born small for the gestational age (SGA) (2100 g.; SDS: −2.28) because of insulin insufficiency, which resulted in neonatal hyperglycemia.

This case underlines that newborns carrying *HNF1b* mutations must be constantly and carefully monitored over time even if born to normo-glycemic mothers to possibly detect the early signs of diabetes. By doing so, pediatricians can provide the most appropriate treatment, thereby preventing the onset of full-blown diabetes and the disease-associated complications. Furthermore, in our case, since infant renal anomalies due to *HNF1b* mutations may progressively deteriorate and evolve into kidney failure, dialysis and, eventually, kidney transplantation cannot be ruled out. In such critical scenario, knowing the clinical history of the glycemic changes presented by the patient can help nephrologists to choose the most appropriate immunosuppressive therapy (corticosteroids and/or immunosuppressive agents), as inappropriate treatment could contribute to accelerating the early onset of diabetes.

## Conclusion

Finally, this case highlights the valuable role of modern biotechnologies in diagnosing challenging medical conditions. Indeed, NGS enabled us to make an absolutely unexpected prenatal diagnosis by allowing us to rapidly identify the *HNF1b* mutation underlying the fetal renal abnormalities detected on prenatal ultrasound and to diagnose a neonatal form of HNF1b dysglycemia.

## Data Availability

Data transparency.
